# Fracture Non-Union: A Review of Clinical Challenges and Future Research Needs

**DOI:** 10.5704/MOJ.1907.001

**Published:** 2019-07

**Authors:** SK Stewart

**Affiliations:** Department of Bioengineering, Imperial College London, United Kingdom

**Keywords:** bone, non-union, fracture, bone healing, biotechnologies

## Abstract

Non-union of bone following fracture is an orthopaedic condition with a high morbidity and clinical burden. Despite its estimated global prevalence of nine million annually, the limit of bone regeneration therapy still results in patients living with pain, a reduced quality of life and associated psychological, social and financial repercussions. This review provides an overview of the current epidemiological and aetiological data, and highlights where the clinical challenges in treating non-union lie. Current treatment strategies are discussed as well as promising future research foci. Development in biotechnologies to treat non-union provides exciting scope for more effective treatment for this debilitating condition.

## Introduction

Skeletal bone has a remarkable capacity for regeneration. The healing of bone after fracture is a phenomenon arising from a complex interplay of mechanical and biological factors. These factors are perfectly orchestrated to bring about consolidation of a fracture in three months, resulting in a functionally sound repair. Impairment of one or more of these factors can result in failure of the bone to heal, a condition termed ‘non-union’.

Though ill-defined, non-union can be considered as the complete cessation of reparative processes of bone healing^1^. Temporal, clinical and radiological parameters are also used to define and diagnose the condition. The American Food and Drug Administration cite non-union as being “established when a minimum of nine months has elapsed since injury and the fracture shows no visible progressive signs of healing for three months”^2^. However, marked variation in these temporal parameters exist in the literature^3-5^.

A clinical finding of the presence of motion and/or pain at the fracture site may be used in the context of assessment and diagnosis of non-union^5,6^. Radiological parameters can also guide the clinician towards the diagnosis of a non-union, with an accepted radiographic criterion of the absence of bridging callus in at least three of the four cortices^7^. However, seldom are all three parameters used in unison with each other and the lack of consensus on definition confers a subjective influence as to when non-union is diagnosed by the clinician^8^.

The aetiology of non-union can be considered as arising from host factors, biological factors and mechanical factors (Table I). Host factors include smoking, age and gender and all can affect the healing capacity of the fracture. Systematic reviews examining the effect of smoking on non-union have demonstrated that smoking was associated with prolonged time to union and smokers were at twice the risk of experiencing fracture non-union, especially in open fracture^9,10^.

**Table I:** The aetiology of non-union classified by host, biological and mechanical factors

Host factors	Biological factors	Mechanical factors
Smoking	Vascular supply	Fracture configuration
Age	Infection	Method of fixation
Gender	Soft tissue coverage	Degree of immobilisation
Alcohol	Degree of bone loss	
Diabetes		
Steroid use		
NSAID use		
Compliance		

The effects of age and gender on non-union are less well understood. A review of studies examining the effect of the patient’s age on non-union identified it as a risk factor in 38 out of 62 (61%) of the studies^11^. Of note, the authors found a variable association between age and non-union depending on the bone fractured: in non-unions of the humerus, there was no significant association between the age and the healing capacity, with a non-union rate of 1.1%^12^. Conversely, age was a significant predictor on the healing capacity of clavicle fractures, demonstrating a non-union rate of 6.2%^13^. The authors concluded that the effect of age may be dependent on the type of fracture sustained, the management chosen, and that age may be a surrogate for the prevalence of other risk factors that potentially increase with age, such as diabetes, and also NSAID use. Moreover, Mills *et al* demonstrated that the non-union rate per fracture in a large adult population was highest in the 30- to 44-year age group, 2.5 times higher than the rate seen in adults aged 75 years and over^14^.

Regarding gender, there is little doubt that women are at increased risk of sustaining a fracture with increasing age, secondary to their predilection of osteoporosis development. What is less clear is whether their fracture healing potential is also compromised. Mills *et al* found that the rate of non-union in a large adult population of fractures was 2.3% for men compared with the lower rate of 1.5% for women^14^. In a similarly designed study, Zura *et al* also demonstrated a marginal difference on rates of non-union between sexes, with men demonstrating a 5.4% risk compared with a 4.6% risk for women^15^. There is a paucity of large scale studies or systematic reviews examining the effect of gender on the risk of non-union. The effect of confounding factors such as whether men undertake higher risk activities, therefore leading to higher energy fractures compared with women, which consequently increases their risk of developing non-union, needs to be evaluated. Further research is required to delineate this relationship.

Biological factors refer to the local environment of the fracture, such as the presence of infection, the extent of bone loss, the vascularity of the bone and the vascularity and quality of the surrounding soft tissues. Mechanical factors relate to the stability of the fracture. Instability at the fracture site leading to excessive strain is the principal mechanical factor resulting in non-union. This can be through inadequate immobilisation, or internal or external fixation, leading to excessive motion at the fracture site.

Two distinct types of non-union are identified radiographically, determined by the amount of new bone forming at the fracture site: atrophic and hypertrophic. Atrophic non-union is associated with inadequate biological factors, and is established in the early stages of fracture healing. Atrophic non-union is typified radiologically by the paucity of callus formation at the fracture site. The bone ends are atrophic with no healing potential. Historically, it was believed that atrophic non-unions demonstrated avascular bone ends, but more recent studies report that atrophic non-unions can be well vascularised^12^.

Conversely, hypertrophic non-union is used to describe non-unions where there is excessive callus formation radiographically. However, the callus formation is disorganised and outwith the fracture site so the fracture remains ununited. It is associated with inadequate mechanical stability, and is established in the later ‘reorganisational’ stages of bone healing. Excessive motion creates initially high strain to the local precursor cells, which are later sensitised by biochemical mediators resulting in activation and proliferation^16^. Hypertrophic non-union is seen on radiographs in three classic configurations depending on the degree of inappropriate mechanical movement at the fracture site. Elephant-foot configurations are a consequence of insufficient fixation, inadequate immobilisation and premature weight-bearing^5^. Ossification occurs at the periphery of the fracture ends giving a characteristic elephant foot appearance. Horse-shoe configurations are less hypertrophic with a smaller amount of callus. Mechanical movement is greater than that seen in elephant foot configurations. Oligotrophic non-unions have minimal amounts of callus in the fracture zone and are a result of significant displacement at the fracture site because of inadequate immobilisation or fixation.

## The Clinical Problem

Epidemiological data of non-union estimate the overall risk of developing non-union following fracture to be 1.94.9%^14,15^. However, this figure varies greatly depending on a number of factors.

The anatomical location of the fracture has a considerable influence on determining the risk of progression to non-union. Tibial fractures have garnered notoriety in the literature for being the long bone with the greatest propensity to progress to non-union^14,15,17^, with rates of non-union reaching as high as 23% in a single case series of tibial fractures^18^. Lowest rates of non-union were reported in a review of 15,249 non-unions for fractures of metacarpals and radii at 1.5% and 2.1% respectively^15^.

Similarly, the mechanism of injury will determine the likelihood of a fracture progressing to non-union. In a meta-analysis exploring the rate of healing in 536 open tibial fractures by Giannoudis and Papakostidis, the average time to healing was 37 weeks (compared with the standard healing length of time of 12 weeks) with a reported non-union rate of 6%^19^. However, in the same study they reported the healing rate of 521 open femoral fractures to be comparable to overall healing rates of fractures at 98%. This emphasises the salient point that the use of the terminology ‘open fracture’ is inadequate without context, such as the associated features of the open injury, contamination risk, soft tissue damage and compromise of a soft tissue envelope.

The cost of treatment of a single bone fracture non-union is high, estimated at between £7000 and £79,000^20-23^. With the UK population approaching 67 million and an individual’s annual fracture risk being 3.6%^24^, even by conservative estimates the annual bill for treating non-union is £320 million. Non-union therefore represents a significant financial and clinical burden both in UK and worldwide.

**Fig. 1:**
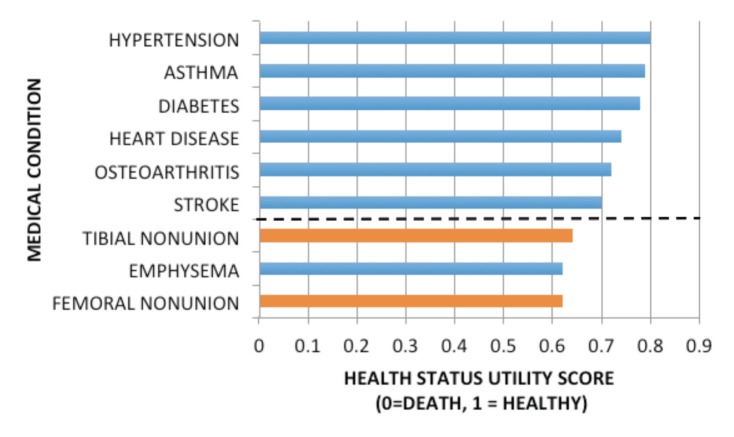
Health Status Utility Scores based on Time Trade Off (0 = death. 1 = perfect health). The dashed line separates the medical conditions associated with a utility score significantly better than femoral non-union (p<0.05). Adapted from Brinker *et al*^25^.

Non-union in most instances is a painful condition, with a marked impact on an individual’s morbidity and quality of life. Brinker *et al* examined the degree of morbidity imposed by non-union, obtaining a utility score based on ‘Time Trade-Off’ (TTO) from 832 patients with a long-bone non-union^25^. The TTO concept describes the percentage of a patient’s life that he or she would be willing to trade to obtain perfect health. The utility score is calculated from subtracting the TTO percentage from ‘1’. Thus, if a patient was willing to trade 30% of life for perfect health, the utility score would be 0.7 (1-0.3). Utility scores therefore range in value from 0 (death) to 1 (perfect health). Brinker *et al* found that those individuals with a femoral fracture non-union had a utility score of 0.62 and those with a tibial non-union had a utility score of 0.68. Moreover, comparing these scores with utility scores taken from TTO scores from patients with other chronic conditions, demonstrated that conditions such as asthma, diabetes, end stage osteoarthritis and stroke had a higher utility score which was statistically significant (Fig. 1). In short, people were willing to trade more years of their life to be rendered free of non-union than for any other medical condition analysed, other than emphysema.

### Current Management

The management of non-union is a clinical challenge to even the most experienced orthopaedic surgeon. The optimal way to treat it has evolved dramatically since the recommendation of the use of a seton in 180226, and is guided by the aetiology of the non-union. Atrophic non-unions where biological factors have driven the failure to heal require adjustment of the biological environment. Equally, hypertrophic non-unions in which mechanical failings have caused the non-union require adjustment of the mechanical environment.

**Fig. 2:**
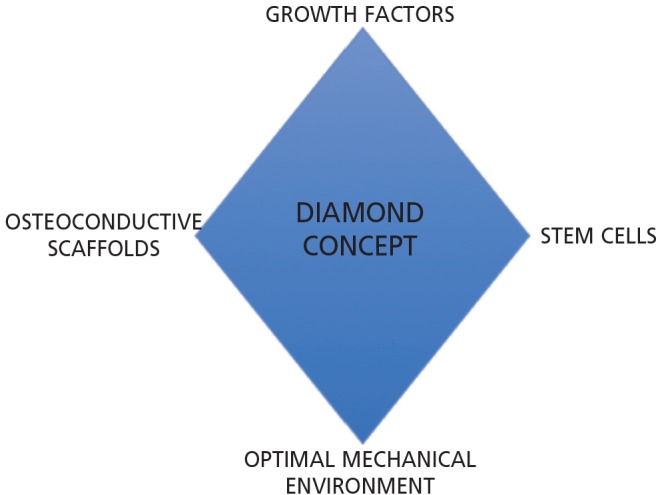
The diamond concept of fracture healing describes the three biological prerequisites (stem cells, growth factors and osteoconductive scaffolds) and one mechanical prerequisite (optimal mechanical environment) required for bone healing to occur.

Use of autologous bone graft (ABG) has dominated the way atrophic non-union has been managed over the past century^27^, owing to its innate capacity to recreate the biological environment of normal bone healing. However, obtaining ABG is not without risks and is associated with donor site morbidity^28^. Recent research has therefore focused on the component parts of ABG, namely mesenchymal stem cells, growth factors and osteoconductive scaffolds. The addition of a favourable mechanical environment to these three biological factors has resulted in the ‘diamond concept’ being coined to describe the optimum conditions for bone healing^29^ (Fig. 2).

### Mesenchymal stem cells

Mesenchymal stem cells (MSCs) are found throughout the adult human body in tissues including bone marrow, peripheral blood, adipose tissue and skin, and possess the two characteristics of any stem cell: (a) the ability to self-renew indefinitely and (b) the ability to differentiate into distinct lineages of mature cells. MSCs have the capacity to differentiate into a number of cell phenotypes, including osteoblasts (bone cells), chondroblasts (cartilage cells) and adipocytes (fat cells). Their differentiation potential and ultimate lineage is determined by mechanical, chemical and hormonal stimuli.

Mechanical stimuli can govern mesenchymal stem cell differentiation through an array of mechanisms, including shear strain, electromagnetic fields, fluid flow and nanodisplacement. Fluid flow has been shown to not only upregulate key genes linked with osteogenesis, but also to increase MSC proliferation^30^. Piezo-driven nanodisplacement can induce osteoblastogenesis in mesenchymal stem cells^31^. Hormonal and chemical signals include cytokines and growth factors. Bone morphogenetic protein-4 has been shown to induce MSCs to become bone and articular cartilage precursors^32^, whereas bone morphogenetic protein-7 is a potent stimulator of MSCs to become bone precursors^33^.

The ability to control MSCs through external signals to induce osteoblastic differentiation has generated much research interest in their use in non-union treatment. Application of shockwaves convey a mechanical stimulus to MSCs through the delivery of pressure, tensile and shearing forces to the cells. This treatment modality referred to as ‘extra-corporeal shock wave therapy’ (ESWT) has been shown to have promising results on the treatment of non-union. A recent review examining eleven peer-reviewed articles utilising ESWT in the treatment of acute long bone fractures and delayed unions and non-unions demonstrated an average union rate of 76%^34^. However, only one of the studies was a randomised controlled trial with the remainder being case series illustrating level four evidence, thus being of insufficient quality to be able to make a recommendation. Furthermore, lack of a standardised definition of non-union and variable treatment regimens renders quantitative analysis difficult, and further stringent clinical trials are warranted.

Similarly, use of low-intensity pulsed ultrasound (LIPUS) waves can confer a mechanical stimulus on mesenchymal stem cells, directing them towards an osteoblastic lineage through conversion of the mechanical signal in to an intracellular chemical signal (a concept referred to as ‘mechanotransduction’). Zura *et al* reported a healing rate of 86.2% on 767 patients with non-unions, in concordance with other studies with smaller cohorts^35-37^. However, these studies are often confounded by a lack of a control group or exclusion of complicated non-union fractures e.g. with associated infection. Further work is therefore required to clearly establish the therapeutic benefit of LIPUS on non-unions.

### Growth Factors

Growth factors describe those naturally occurring substances which are capable of stimulating cellular growth and proliferation. They include bone morphogenetic proteins (BMP), transforming growth factor β (TGFβ), vascular endothelial growth factor (VEGF) and platelet derived growth factor (PDGF). Of these, BMPs have been the most intensely studied. First identified in the 1960s as a potent bone inductive agent^38^, 18 different BMPs have been discovered with two (recombinant human BMP-2 and BMP-7) licensed for commercial use in orthopaedic applications^39,40^.

BMPs exert their osteogenic action through their interaction with mesenchymal stem cells, binding to surface receptors and subsequently triggering intracellular canonical pathways, the most pertinent being the Smad transcription factor pathway^41^. Activation of this signalling pathway ultimately leads to upregulation of transcription factors strongly associated with osteoblastic differentiation such as RUNX2 and OSX. It is through activation of these transcription factors that BMPs exert their osteogenic effect, by inducing markers specific to osteoblast differentiation including osteocalcin and osteocalcin.

BMPs have been extensively studied both in vitro and in vivo and have shown great potential in both regenerative medicine fields and for the treatment of bone conditions such as open fractures and non-union^39,40,42,43^. However, there has also been concern about their side-effect profile, including ectopic bone development, haematomas in soft tissues and resorption around implants^44^. A recent systematic review examining their clinical effect on bone healing concluded that their osteogenic effect in a clinical setting is inconclusive, and further research utilising better designed, comparative studies is mandated to fully optimise the therapeutic use of BMPS in conditions such as non-union^45^.

### Osteoconductive Scaffolds

Osteoconductive scaffolds provide the physical framework through which osteoinductive agents such as MSCs and GFs can be delivered to the site of the non-union. Scaffolds can derive from naturally occurring bone or from synthetic substitutes. Natural scaffolds can be either autogenic or allogenic, and can be in the form of demineralised matrix, cancellous and cortical, corticocancellous, osteochondral and whole bone segments^46^. Synthetic scaffolds are typically made from β-tricalcium phosphate, hydroxyapatite or collagen. They have the advantage of avoiding risks of infection, immunogenicity and rejection secondary to their acellular construct, but have the disadvantage of being less osteoconductive than natural constructs.

Rarely will these three biological factors be used in isolation, particularly in the clinical setting. Rather permutations of either two or all three elements are used in either a preclinical or clinical setting to recreate the efficacy of ABG in the treatment of non-union^47-49^. This has been termed the ‘polytherapy approach’^50^. Healing rates can be further augmented by the addition of one or more of these biological factors to ABG. Enhancement of ABG with BMP-7 was associated with a success rate of 100% in a series of 45 of long bone non-unions^51^, whereas in other studies the addition of BMP-7 to ABG was found to be superior at healing non-unions than ABG alone^52,53^.

### Mechanical Environment

Optimisation of the mechanical environment represents the cornerstone of hypertrophic non-union management, as well as being a key tenet of atrophic non-union healing. Excessive strain generated through inadequate immobilisation or suboptimal fixation is primarily responsible for the formation of hypertrophic non-union. Unwanted movement at the fracture prevents the transformation from fibrous tissue at the fracture site to osseous tissue. Correction of the strain level, either through revision surgery or reimmobilisation, acts to restore mechanical stability. This results in calcification of the fibrous cartilage, which can only then be penetrated by new vessels, allowing bony bridging and remodelling of the non-union site^16^.

## Future Research Foci and Needs

### Mechanotransductive Agents

Despite the advances in the management of non-union that have been borne out of research and technology, there still exists an undefined consensus regarding the optimal management choice for the condition. One of the most exciting and rapidly expanding areas of research to aid bone healing is mechanotransductive technologies. Mechanotransduction describes how an external physical stimulus can induce a biological response at a cellular level. The pathways involved are complex and a more detailed review of the processes can be read elsewhere^54^. Fundamentally however, detection of local physical forces by specific cell receptors brings about changes in intracellular signalling pathways, ultimately altering the cell’s phenotype and function.

The ability of MSCs to differentiate in a number of cell lineages including osteoblasts has been harnessed by mechanotransductive technologies focussed on bone regeneration. External physical stimuli including hypergravity, shear forces and compressive loading have all been shown to be inducers of MSC differentiation^55^. Exposure of rat MSCs to a hypergravity environment induced upregulation of osteogenic markers both at protein and gene level, as well as changes to cell morphology and shape suggesting enhancement of the osteogenic differentiation of MSCs^56^. Application of shear stress by oscillatory fluid flow has been demonstrated to induce commitment of MSCs to an osteogenic lineage in a number of studies^30,57,58^. The physical forces generated from electromagnetic fields have also been shown to effectively induce osteogenic differentiation of MSCs^59-61^. Today however, there exists a paucity of studies employing these mechanotransductive technologies in an in vivo model of non-union. Translatability of these promising techniques is critical to better understanding their applicability in a clinical setting for the patient with a debilitating non-union.

### Platelet Rich Plasma

Platelet rich plasma (PrP) is also garnering much attention for its potential bone healing properties. The believed efficacy of PrP derives from the knowledge that platelets are present in the early ‘inflammatory’ stages of fracture healing. Migration of platelets to the fracture site to form the haematoma plug is twinned with the secretion of cytokines including growth factors, haemostatic factors and adhesion molecules, the most relevant being PDGF, VEGF, BMPs, TGF β and insulin-like growth factor (IGF). Furthermore, platelets promote angiogenesis and recruit MSCs^62,63^. The increased concentration of platelets within PrP is therefore understood to deliver a superadded effect to the bone healing process. Its appeal is further enhanced by the non-invasive method through which it is obtained, taking just a peripheral blood sample from the patient in contrast to the morbidity associated with obtaining a bone marrow aspirate. Animal studies utilising PrP on long bone defect models show promising evidence, with a recent systematic review demonstrating a 100% increase in bone formation on radiographs where PrP was utilised^64^. However, other systematic reviews have found less convincing results, with quality of evidence being hampered by unspecified platelet concentrations and a paucity of randomised controlled trials^65-67^.

Furthermore, the clinical characterisation of PrP will always be challenging given the inherent heterogenic nature of samples from individual patients. Many studies also analysed the effect of PrP with an adjuvant therapy such as MSCs and scaffolds, making the case for the efficacy of PrP difficult to delineate. What is clear is a need for a standardised preparation protocol for PrP in conjunction with more robust research trials employing RCTs, in order to better elucidate the capacity to use PrP in a clinical setting for non-union.

### Chitosan

Chitosan, derived from the exoskeleton of crustaceans, is a polysaccharide that provides a microenvironment for cell proliferation and extracellular matrix production, as well as possessing osteoinductive characteristics^68^. Its osteogenic effect is exerted through stimulation of growth factors, differentiation and cell aggregation in the wound, thereby promoting and accelerating the regeneration of bone tissue^69^. Mesenchymal stem cells treated in vitro with chitosan demonstrate upregulation of osteogenic genes and calcium mineralisation^70-72^. Chitosan’s appeal is further enhanced by its ability to be delivered in various forms including as a paste or powder, as well as its possession of antimicrobial properties^73^.

Although no human trials have yet to evaluate chitosan as a treatment modality for non-union, a number of animal studies have utilised chitosan either alone or in conjunction with another therapy in models of non-union. A recent systematic review examining pre-clinical therapies to prevent or treat non-union identified chitosan as a promising osteogenic agent^74^. Four out of six papers that utilised chitosan as a treatment for an animal model of non-union found that chitosan was superior to a control treatment^75-78^. However, three out of these four papers found that delivery of the chitosan with an adjuvant treatment improved the efficacy of the bone healing above chitosan alone.

The use of chitosan in the clinical sphere is less well documented. Although its bone regenerative properties is gaining favourability in dentistry^79^, chitosan’s efficacy in fracture non-unions has yet to be ascertained. However, the encouraging results seen in animal studies provide evidence that translatability of its application to clinical trials is warranted.

## Conclusion

Bone healing is a complex interplay of a number of factors, all carefully orchestrated through multiple pathways. Healing a fracture non-union represents trying to replicate this bone healing process in its most difficult environment. What is evident from current therapeutic regimens and promising evolving research is that there may never be a single agent that can replace the body’s innate resources. However, a number of current and new therapies demonstrate significant osteogenic capabilities. Harnessing these in the most appropriate manner and twinning them with other agents to replicate the ‘diamond’ concept is likely to yield the most promising therapies. Furthermore, coordination between the research community to ensure treatment modalities are reproducible, validated and comparable gives us the greatest chance of solving the perennially difficult clinical challenge of bone non-union.
